# The Diagnostic Performance of Ultrasonography in the Evaluation of Extrathyroidal Extension in Papillary Thyroid Carcinoma: A Systematic Review and Meta-Analysis

**DOI:** 10.3390/ijms24010371

**Published:** 2022-12-26

**Authors:** Peter P. Issa, Aaron L. Albuck, Eslam Hossam, Mohammad Hussein, Mohamed Aboueisha, Abdallah S. Attia, Mahmoud Omar, Seif Abdelrahman, Gehad Naser, Robert D. E. Clark, Eman Toraih, Emad Kandil

**Affiliations:** 1 School of Medicine, Louisiana State University Health Sciences Center, New Orleans, LA 70112, USA; 2 School of Medicine, Tulane University, New Orleans, LA 70112, USA; 3 Surgical Oncology Department, National Cancer Institute, Cairo University, Cairo 11796, Egypt; 4 Genetics Unit, Department of Histology and Cell Biology, Faculty of Medicine, Suez Canal University, Ismailia 41522, Egypt

**Keywords:** ultrasonography, papillary thyroid carcinoma, extrathyroidal extension, meta-analysis

## Abstract

Extrathyroidal extension (ETE) in patients with papillary thyroid carcinoma (PTC) is an indication of disease progression and can influence treatment aggressiveness. This meta-analysis assesses the diagnostic accuracy of ultrasonography (US) in detecting ETE. A systematic review and meta-analysis were performed by searching PubMed, Embase, and Cochrane for studies published up to April 2022. The pooled sensitivity, specificity, and diagnostic odds ratio (DOR) were calculated. The areas under the curve (AUC) for summary receiver operating curves were compared. A total of 11 studies analyzed ETE in 3795 patients with PTC. The sensitivity of ETE detection was 76% (95%CI = 74–78%). The specificity of ETE detection was 51% (95%CI = 49–54%). The DOR of detecting ETE by US was 5.32 (95%CI = 2.54–11.14). The AUC of ETE detection was determined to be 0.6874 ± 0.0841. We report an up-to-date analysis elucidating the diagnostic accuracy of ETE detection by US. Our work suggests the diagnostic accuracy of US in detecting ETE is adequate. Considering the importance of ETE detection on preoperative assessment, ancillary studies such as adjunct imaging studies and genetic testing should be considered.

## 1. Introduction

Papillary thyroid carcinoma (PTC) is the most common type of thyroid cancer and, with respect to prevalence, is one of the fastest growing cancers in the United States [1]. This is largely due to increased detection and appears to be the result of widespread and increased use of highly sensitive diagnostic tests and imaging modalities such as ultrasonography (US) and computed tomography (CT) [2]. Extrathyroidal extension (ETE) is an important parameter which can be assessed by imaging studies and which has been associated with an increased risk in mortality for patients diagnosed with PTC [3]. The 15-year survival rate of patients with PTC who also present with ETE during the course of their disease has been shown to be significantly lower than that of patients without ETE [4]. 

According to the American Joint Committee on Cancer (AJCC) TNM Staging for Thyroid–Differentiated and Anaplastic Carcinoma (8th Edition, 2017), ETE is divided into either minimally invasive or gross ETE. Minimal ETE refers to the extension of the primary tumor to only the surrounding peri-thyroid soft tissues, while gross ETE implies that the primary tumor has invaded surrounding musculature, the trachea, larynx, vasculature, and/or the esophagus [5]. The current American Thyroid Association (ATA) guidelines recommend extensive surgery (e.g., total thyroidectomy) for PTC with ETE or nodal disease, but also acknowledge active surveillance as an appropriate alternative in the absence of ETE [6]. Although active surveillance for thyroid cancer has gained acceptance, as reflected in the ATA guidelines, many clinicians in the United States are still skeptical. One reason for this hesitancy is the accuracy of detecting tumor progression by imaging studies, which may be limited in their reliability in identifying concerning features. Therefore, it is critical to employ imaging modalities that accurately identify tumors that present high-risk features with a high likelihood of progression. Notably, a delay in surgery for thyroid cancer is associated with a 94% higher chance of mortality [7], underscoring the importance of successfully identifying the need for surgical intervention via US imaging.

US is the standard mainstay imaging modality for both the detection and diagnosis of PTC [8]. Still, US has been shown to be limited in assessing the extent of ETE [9]. Evaluating the sensitivity and specificity of US is of utmost importance to healthcare teams aiming to assess ETE in patients with PTC. Doing so will better assist clinicians and surgeons in patient risk stratification and surveillance of disease progression. This meta-analysis aims to evaluate the diagnostic accuracy of US in the detection of ETE in patients with PTC.

## 2. Methods

### 2.1. Search Strategy 

This meta-analysis was conducted in compliance with the Preferred Reporting Items for Systematic Review and Meta-Analyses (PRISMA) guidelines [10]. A search for published primary studies which investigated the diagnostic accuracy of ultrasound in detecting ETE was conducted in April 2022. The databases PubMed, Embase, and Scopus were searched utilizing the following terms: “thyroid” AND “extrathyroidal extension” OR “extrathyroidal” AND “ultrasonography” OR “sonography” OR “ultrasound”. Only works published in the English language were considered. 

### 2.2. Study Selection

All results from the search query were subject to the inclusion and exclusion criteria of this study. The inclusion criteria were (1) randomized controlled trials, cohort studies, or case-control studies which (2) reported the accuracy of US in detecting ETE (3) in patients who had PTC. Finally, all studies must have confirmed the presence or absence of ETE on post-operative specimen surgical pathology. Abstracts, case reports, letters, and works which were not primary studies (including systematic literature reviews and clinical reviews) were excluded. 

All results of the search query were subject to screening. All articles which met the inclusion criteria were subsequently subject to data extraction. Parameters collected included basic study characteristics such as study year, author, title, country, institution, study period, and study design. In addition, study sample size and parameters relevant to determining sensitivity and specificity were collected, including the count of true positives, true negatives, false positives, and false negatives. 

### 2.3. Data Abstraction

The screening and data extraction were conducted by two independent investigators (P.P.I. and A.L.A.). Any inconsistencies in the study screening or data extraction were settled by a senior author (M.H.). Data were extracted into a predesignated excel sheet. Data extracted included the author’s name, date of publication, journal name, study type, and number of patients, as well as outcomes of interest, including true positive, true negative, false positive, and false negative counts.

### 2.4. Statistical Analysis 

Statistical analysis was conducted using MetaDisc1.4 software (Unit of Clinical Biostatistics, Madrid, Spain). Sensitivity, specificity, negative and positive likelihood ratios (LR), diagnostic odds ratio (DOR), and a 95% confidence interval (CI) were estimated. The area under the curve (AUC) was generated. The comparison between sub-groups was performed using student’s *t*-test. To determine if certain parameters may have been influencing the accuracy of the lymph node metastasis (LNM) detection, we conducted sub-group meta-regression analyses. Since neither US probe frequency nor body mass index (BMI) was consistently reported in our study cohort, we analyzed study publication year (≥2015 versus <2015) as a potential proxy for technological advancement and country (United States versus other) as a potential proxy for patient body habitus. We quantified the heterogeneity using the I-square (I^2^) and Chi-squared tests. A fixed-effects model was used to analyze pooled results. However, in the presence of heterogeneity as evidenced by I^2^ > 50% or *p* < 0.05, a random-effects model was used. A meta-regression model was conducted to trace putative sources of heterogeneity according to the study characteristics (study design, sample size, and the year of publication). 

## 3. Results

### 3.1. Literature Search & Study Population

**Figure 1:**
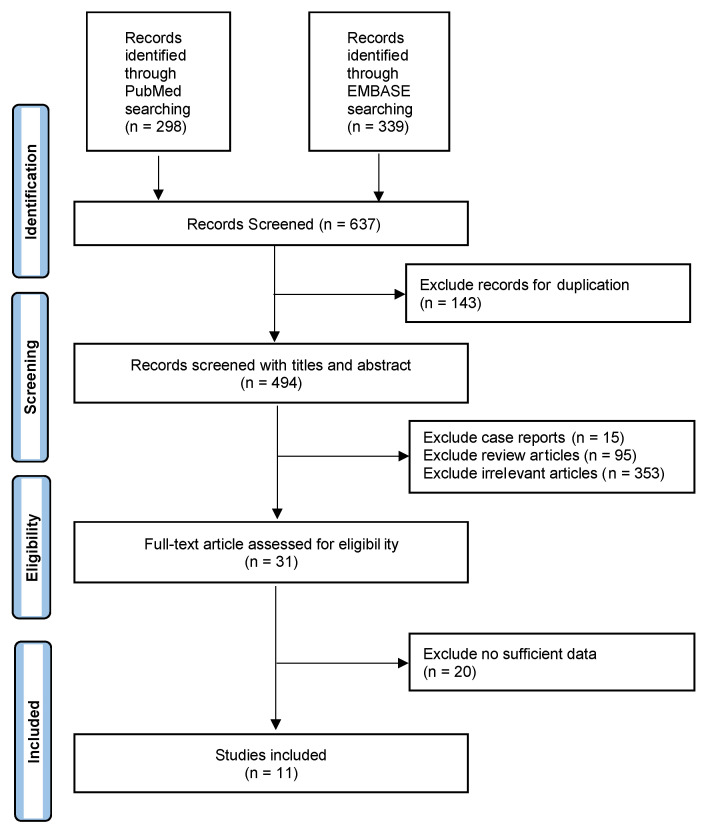
Workflow of the literature search for the diagnostic performance of ultrasound in detecting extrathyroidal extension.

Our search query elicited 494 unique articles (637 total, 143 duplicated). A total of 483 articles did not meet the inclusion criteria, allowing an analysis of eleven unique primary studies. The workflow of the included studies is shown in Figure 1. The studies took place from 2014 until 2020 and represent works from multiple countries, including five from Korea, three from China, two from the United States, and one from Italy. Of the eleven studies, four were prospective in study design. A total of 3795 patients were included in the study. The characteristics of the studies included are shown in Table 1. All diagnoses of the extrathyroidal extension were confirmed on post-operative surgical pathology.

### 3.2. Detection of Extrathyroidal Extension by Ultrasound

A total of eleven studies analyzed 3795 patients with PTC. The sensitivity of ETE detection was 76% (95%CI = 74–78%). The specificity of ETE detection was 51% (95%CI = 49–54%). The DOR of detecting ETE by US was 5.32 (95%CI = 2.54–11.14). The AUC of ETE detection was determined to be 0.6874 ± 0.0841. Table 2 provides a summary of the diagnostic accuracy of US in detecting ETE. 

### 3.3. Detection of Extrathyroidal Extension Sub-Group Analyses & Meta-regression

To determine whether parameters could be influencing the relatively low detection accuracy of ETE by US, we conducted sub-group analyses (Table 3). When sub-grouped by year of publication (≥2015 vs. <2015), there was a general trend toward increased diagnostic accuracy. ETE detection specificity, sensitivity, and DOR prior to 2015 was 69.7% (95%CI = 65.2–74%) and became 78.9% (95%CI = 76.5–81.2%); 41.6% (95%CI = 36.2–47.2%) and became 53.9% (95%CI = 50.9–56.8%); and 5.077 (95%CI = 1.182–21.812) and became 5.569 (95%CI = 2.305–13.455), respectively. With respect to study design, studies that were retrospective in nature tended to have better sensitivity (63.9%, 95%CI = 60.5–67.2% vs. 89.2%, 95%CI = 86.9–91.3%) and DOR (1.917, 95%CI = 0.735–5.004 vs. 10.423, 95%CI = 5.074–21.41), but similar specificity (51.8%, 95%CI = 47.8–55.7% vs. 50.8%, 95%CI = 47.3–54.2%). When sub-grouped by country, the specificity of studies conducted in China, Korea, and the USA were 67.7% (95%CI = 63.2–71.9%), 75.3% (95%CI = 72.1–78.4%), and 87.2% (95%CI = 83.2–90.4%), respectively. The sensitivity of studies conducted in China, Korea, and the USA were 62.7% (95%CI = 57.2–67.8%), 41.6% (95%CI = 37.9–45.4%), and 63.1% (95%CI = 57.8–68.2%), respectively. With respect to DOR, the USA tended to have a higher estimate at 10.8695 (95%CI = 4.207–83.078) when compared to China (5.043, 95%CI = 0.0838–30.368) and Korea (2.452, 95%CI = 0.946–6.356). 

Independent predictors of ETE detection by US were analyzed, including the country in which the study was conducted (United States versus Asia), the design of the study (prospective versus retrospective), and the year of the study (≥2015 versus <2015) (Table 4). Study country (*p* = 0.58), study design (*p* = 0.1), and study year of publication (*p* = 0.54) did not influence ETE imaging detection accuracy.

## 4. Discussion

Preoperative assessment of patients with PTC is imperative for appropriate surgical planning. ETE is an important parameter collected on preoperative ultrasound which significantly alters patient prognosis. Since patient T/N/M staging is typically of more importance than malignancy grading in patient prognosis, preoperative assessment of ETE has been shown to significantly influence patient survival [21,22]. Our meta-analysis found that ultrasound in general was beneficial, but only a moderate imaging study choice with respect to detecting ETE. 

The incidence of ETE in thyroid cancer varies between 5–45% according to the current literature [23]. Though patients with PTC typically have a >95% 10-year survival rate and an excellent prognosis, the presence of ETE on preoperative assessment is a reliable predictor of disease progression. A 2018 study of patients with PTC greater than 1 cm found that those who had ETE were significantly more likely to present with lymph node metastasis (67.4% vs. 33.3%, *p* < 0.001) and capsule invasion (93.8% vs. 25.0%, *p* < 0.001), and receive radioactive iodine ablation therapy (97.7% vs. 88.9%, *p* < 0.001). In patients with ETE and papillary thyroid microcarcinomas (PTC < 1 cm), they found similar results, reporting increased rates of lymph node metastasis (34.3% vs. 24.1%, *p* < 0.001), capsule invasion (97.2% vs. 25.0%, *p* < 0.001), and radioactive iodine ablation therapy (97.2% vs. 80.6%, *p* < 0.001) [24]. Therefore, the presence of ETE can serve as a reliable prognostic parameter regardless of carcinoma size. By the current ATA guidelines, PTC patients with ETE are classified as having stage 3 cancer [6]. In these patients, complete surgical resection is imperative for optimal patient prognosis [6]. Therefore, accurate preoperative assessment is important. 

Ultrasound is widely considered the mainstay and first-line method for evaluating and characterizing thyroid nodules. US is a readily available, relatively low-cost, and quick imaging study that imparts no radiation [25]. Conversely, however, ultrasound is also an operator-dependent imaging study which varies in accuracy from user to user and patient to patient [25,26,27]. Several studies have reported the diagnostic accuracy of ETE detection on ultrasound. A recent 2020 study using a “nonrestrictive definition” (i.e., the nodule abuts the thyroid capsule with or without signs of disruption) for assessing ETE found a sensitivity of 86.4%, specificity of 29.8%, and DOR of 2.68 [12]. Similarly, our meta-analysis found a sensitivity of 76.4%, specificity of 51.2%, and DOR of 5.317. Of note, when the same study utilized a “very restrictive” definition for ETE (i.e., the nodule disrupts the capsule and invades surrounding tissues), they found a specificity of 100%, DOR of 14.25, but a sensitivity of only 6.8% [12]. Ultrasonographers, surgeons, and radiologists alike should be aware of this tradeoff and realize this limitation of US as an imaging modality. Moreover, although most studies did not stratify their data in this manner to allow for analysis, it is worth mentioning that, similar to the effect of the stringency of the definition of ETE, ETE can be classified as minimal or gross. ETE classified as minimal refers to minimal extension of the primary tumor, only into and around the surrounding peri-thyroid soft tissues. Gross ETE refers to gross extension of the primary tumor, into and around the trachea, larynx, surrounding musculature and vasculature [5], and is understandably easier to detect by ultrasound. A recent 2021 study investigated the diagnostic accuracy of US in 305 differentiated thyroid cancer patients and stratified their findings based on post-operative ETE histology (minimal or gross) to demonstrate a difference in detection accuracy. The authors reported a sensitivity of 30%, specificity of 93%, and accuracy of 76% in those with minimal ETE, but a sensitivity of 78%, specificity of 99.7%, and accuracy of 98% in those with gross ETE [28]. Taken together, clinicians and surgeons should recognize the tradeoff and uncertainty in detecting ETE on preoperative ultrasound. Considering this, ancillary testing such as adjunct imaging studies and genetic testing should be considered. 

Ultrasound has been described as an inconsistent imaging modality, depending on the ultrasound technology, the ultrasonographer, and the patient. For example, the prevalence of thyroid nodules was estimated to be 33% in the normal population when using a 7.5 MHz probe [29], but soared to 68% in a study using a 13 MHz probe [30]. Similarly, whether the ultrasound was read and performed by a radiologist or non-radiologist (such as a surgeon or ultrasound technician) may potentially influence detection accuracy [31,32]. A recent meta-analysis including 25 studies and 5768 patients found that preoperative ultrasound read by radiologists and non-radiologists detected lymph node metastasis with similar sensitivity (radiologist: 58% vs. non-radiologist: 62%) and specificity (radiologist: 86% vs. non-radiologist: 78%) [27]. Interestingly, patient body habitus has been suggested to influence ultrasound accuracy. For example, *Choi* et al. reported in 2020 that ultrasound accuracy decreased when comparing non-obese patients (BMI < 30) and obese (BMI ≥ 30) patients. Specifically, the authors reported a sensitivity of 59% which dropped to only 19% in their detection of hepatocellular carcinomas [16]. With respect to the thyroid, a similar phenomenon was recently demonstrated by *Omar* et al. in 2022. In 204 PTC patients, they found the AUC of ultrasound in detecting ETE in non-obese (<30 BMI) patients was 0.71 ± 0.06 which fell in obese (>30 BMI) patients to 0.43 ± 0.05 (*p* = 0.001) [26]. The inconsistency in reporting of patient BMI in the studies included hindered the analysis of these potentially influencing factors, and the authors acknowledge this as a limitation of this study. Therefore, proxies of these variables were analyzed, including study country as a proxy for patient BMI and study publication year as a proxy for ultrasound technology. In our analysis, these factors did not influence the diagnostic accuracy of US in detecting ETE. The authors acknowledge that these proxies are not well-accepted in the literature, but have provided them nonetheless. 

One potential approach to increasing the diagnostic accuracy of detecting ultrasonographic features such as ETE on US is the use of radiomics. Radiomics is the use of machine-learning applied to US imaging to increase diagnostic accuracy. Several works have reported the use of US-related machine learning in assisting benign versus malignant judgement [33], cervical lymph node staging [34], and even BRAF mutation detection [35]. Because it is a novel field, few works have reported the use of radiomics in detecting ETE in PTC patients, including *Wang* et al. (2021) who reported an AUC of 0.837 [36], which is up from our reported 0.6874. Their nomogram included multiple parameters including the location of the nodule, subjective ETE assessment, and their radiomic signature. Their findings suggest that machine-learning in US may be a reliable tool to predict ETE and may warrant further study.

Beyond ultrasound, there are several other imaging modalities used to image the neck in assessment of thyroid pathology. For example, CT is a reliable and consistent imaging study which allows visualization of the neck in 3 dimensions [37]. Defining ETE as more than 25% contact with the capsule, *Lee* et al. found an 87.8% sensitivity and 48.6% specificity using CT [37]. Similarly, magnetic resonance imaging (MRI) has been shown to detect ETE as well. One study reporting on 75 patients with PTC found a sensitivity of 88.7%, specificity of 77.5%, and an accuracy of 83.2% [19]. *Hu* et al. reported that, in patients with PTC, dual imaging of MRI + US was more effective than either US or MRI alone [38]. The authors reported that diagnostic accuracy improved from 80.4% and 79.1% using ultrasound alone or CT alone, respectively, to 96.2% (*p* = 0.001) when used together. Considering the significant improvement in utilizing ancillary imaging studies, and the already only moderate diagnostic accuracy in detecting ETE by US alone, clinicians and surgeons should consider adjunct imaging studies to improve patient risk stratification. 

In addition to adjunct imaging, ETE may be better assessed using molecular markers of cancer. Genotyping was introduced into the latest ATA guidelines to better assist in risk stratification, but its use can potentially predict specific features of advanced thyroid cancer. For example, a common and well-established mutation implicated in thyroid cancer oncogenesis is BRAFV600E mutation. BRAF mutation is prevalent in up to 51% of PTCs [39,40]. A meta-analysis including 22 studies which investigated ETE (N = 4668 patients) found that patients with BRAF mutation were 2.60 times (OR = 2.60, 95%CI = 2.27–2.99) as likely to present with ETE than those without BRAF mutation [41]. Therefore, in patients with PTC which are potentially displaying ETE, determination of BRAF-mutation status may significantly increase the likelihood of accurate assessment. Similarly, telomerase reverse transcriptase (TERT) is another commonly mutated gene in the field of thyroidology. TERT assists in the elongation of telomeric DNA, and its mutation assists in oncogenesis by allowing infinite cell proliferation potential [42]. In patients with PTC, a meta-analysis found that TERT promotor mutation increased the risk of ETE almost two-fold (OR = 1.98, 95%CI = 0.96–4.07) [43]. Interestingly, a meta-analysis of 13 studies (N = 4347 patients) found that the odds of ETE were significantly greater when both BRAF and TERT promotor mutations were present than when either was present alone [44]. BRAF alone conferred a 2.55 increased risk (95%CI = 1.99–3.03) of ETE, but combined (BRAF + TERT) mutation increased the risk eight-fold (OR = 8.14, 95%CI = 5.55–11.94) [44]. The study found similar results with increased odds of advanced TNM staging, lymph node metastasis, and distant metastasis [44]. These findings were recently supported by another meta-analysis by *Zhao* et al. (26 studies, N = 8388 patients) which ranked co-existent BRAFV600E + TERT mutations highest in advanced disease and ETE [45]. Specifically, BRAFV600E + TERT mutations increased the odds of ETE by almost six-fold (OR = 5.80, 95%CI = 3.89–8.64), BRAFV6000E alone by almost two-fold (OR = 1.88, 95%CI = 1.42–2.49), and TERT alone by almost two-fold (OR = 1.72, 95%CI = 1.10–2.68). They found that RAS mutation alone did not increased the odds of ETE on presentation (OR = 0.88, 95%CI = 0.40–1.94) [45]. RAS mutations (including its variants, including NRAS, KRAS, and HRAS) are the most commonly mutated genes in thyroid cancer. In a study using The Cancer Genome Atlas, *Park* et al. reported that RAS mutation was negatively correlated with the occurrence of ETE (OR 0.3, *p* = 0.001) [46]. Similarly, a study genotyping 56 thyroid carcinomas with ETE but without nodal metastasis on presentation found that only two patients (3.6%, 2/56) had RAS mutations (specifically, one classic PTC and one poorly-differentiated thyroid carcinoma) [47]. Considering these common gene mutations, it appears that BRAFV600E and TERT mutations increase the likelihood of ETE in PTC patients but RAS does not. 

Beyond ancillary imaging studies and molecular genetic testing, the determination of ETE by intraoperative frozen section has been demonstrated as a reliable method of ETE determination. In a study of 54 patients with PTC and surgical-pathology confirmed ETE (study of 268 total PTC patients), Park et al. found that ETE was accurately determined by frozen section in 53 patients (53/54, 98.1%) [48]. The authors reported a sensitivity of 66%, specificity of 99%, and positive predictive value of 98% [48]. Determination of ETE intraoperatively by frozen section may therefore be the most reliable method of determining ETE prior to surgery completion. Intraoperative assessment alone, however, without the use of frozen specimen, is of limited use in predicting advanced disease such as ETE or lymph node metastasis [49,50]. 

This study is not without limitation. First, the authors recognize that ultrasound accuracy may be dependent on the qualification of the sonographer (radiologist, surgeon, or ultrasound technician) and the ultrasound technology itself, such as the probe transducer frequency, as well as the patient. Inconsistencies in study reporting of such variables hindered these analyses, though potential proxies were estimated instead. Though most studies were retrospective in nature, lending to potential biases, they took place in many countries and allowed for both a large sample size and diverse study population. However, differences in training qualifications exist between different countries and must be considered.

## 5. Conclusions

The diagnostic accuracy of US in detecting ETE was adequate, with a sensitivity of 76.4%, a specificity of 51.2%, and a DOR of 5.317. Considering the importance of ETE detection on preoperative assessment, ancillary studies such as adjunct imaging studies and genetic testing should be considered.

## Figures and Tables

**Table 1:** Characteristics of the studies included. The study period is reported as months.year.

Study	Year	Study Period	Country	Study Design	Patients
Hu, 2020 [11]	2020	05.2014–12.2018	China	Retrospective	225
Ramundo, 2020 [12]	2020	11.2015–05.2019	Italy	Retrospective	128
Jiao & Zhang, 2017 [13]	2017	10.2011–07.2014	China	Retrospective	166
Yi, 2016 [14]	2016	05.2011–12.2011	Korea	Prospective	61
Kamaya, 2015 [15]	2015		USA	Retrospective	129
Choi, 2014 [16]	2014	12.2012–04.2013	Korea	Prospective	625
Gweon, 2014 [17]	2014		Korea	Prospective	79
Kim, 2014 [17]	2014	01.2011–05.2012	USA	Retrospective	75
Lee, 2014 [18]	2014	05.2009–12.2010	Korea	Retrospective	568
Lee, 2014 [19]	2014	01.2006–12.2012	Korea	Retrospective	252
Wei, 2014 [20]	2014		China	Prospective	317

**Table 2:** Detection of extrathyroidal extension by ultrasound.

	Estimate [95% CI]
**Sensitivity**	76.4% [74.3–78.5%]
**Specificity**	51.2% [48.6–53.8%]
**DOR**	5.317 [2.538; 11.139]
**AUC**	0.6874 ± 0.0841

Data are reported as estimates (95% confidence interval) or estimate ± standard error. CI: confidence interval. DOR: diagnostic odds ratio. AUC: area under the curve.

**Table 3:** Meta-regression analysis of parameters influencing extrathyroidal extension detection accuracy on ultrasound.

Sub-Group			Estimate	95%CI Lower	95%CI Upper
**Year of Publication**	Sensitivity	<2015	78.9%	76.5%	81.2%
		≥2015	69.7%	65.2%	74.0%
	Specificity	<2015	53.9%	50.9%	56.8%
		≥2015	41.6%	36.2%	47.2%
	DOR	<2015	5.569	2.305	13.455
		≥2015	5.077	1.182	21.812
**Study design**	Sensitivity	Retrospective	89.2%	86.9%	91.3%
		Prospective	63.9%	60.5%	67.2%
	Specificity	Retrospective	50.8%	47.3%	54.2%
		Prospective	51.8%	47.8%	55.7%
	DOR	Retrospective	10.423	5.074	21.41
		Prospective	1.917	0.735	5.004
**Country**	Sensitivity	China	67.7%	63.2%	71.9%
		Korea	75.3%	72.1%	78.4%
		USA	87.2%	83.2%	90.4%
	Specificity	China	62.7%	57.2%	67.9%
		Korea	41.6%	37.9%	45.4%
		USA	63.1%	57.8%	68.2%
	DOR	China	5.043	0.838	30.368
		Korea	2.452	0.946	6.356
		USA	18.695	4.207	83.078

DOR: diagnostic odds ratio. CI: confidence interval.

**Table 4:** Meta-regression analysis of parameters influencing extrathyroidal extension detection accuracy on ultrasound.

	Variable	Coefficient	SE	*p*-Value	DOR	95%CI Lower	95%CI Upper
**Study design**	Prospective vs. Retrospective	−1.543	0.806	0.1	0.21	0.03	1.54
**Year of Publication**	≥2015 vs. <2015	−0.505	0.782	0.54	0.6	0.09	4.1
**Country**	USA vs. Asia	−0.098	0.165	0.58	0.91	0.57	1.43

DOR: diagnostic odds ratio. CI: confidence interval.

## Data Availability

Data are contained within the article.
